# Updates on p53: modulation of p53 degradation as a therapeutic approach

**DOI:** 10.1038/sj.bjc.6604098

**Published:** 2008-01-08

**Authors:** A Dey, C S Verma, D P Lane

**Affiliations:** 1 Department of Cell Cycle Control, Institute of Molecular and Cell Biology, 61, Biopolis Drive, 03-05 Proteos, Singapore 138673, Singapore; 2 Bioinformatics Institute, 21 Biopolis Way, 07-01 Matrix, Singapore 138668, Singapore

**Keywords:** p53, Mdm2 (Hdm2), Mdm4 (MdmX, Hdm4, HdmX), small molecule inhibitors

## Abstract

The p53 pathway is aberrant in most human tumours with over 50% expressing mutant p53 proteins. The pathway is critically controlled by protein degradation. Here, we discuss the latest developments in the search for small molecules that can modulate p53 pathway protein stability and restore p53 activity for cancer therapy.

p53 is probably the most extensively studied tumour suppressor protein with a critical role in controlling cell cycle arrest and apoptosis (Vogelstein *et al*, 2000; Vousden and Lane, 2007). The p53 protein acts as a highly regulated sequence-specific DNA-binding protein that in response to a wide variety of stress signals undergoes post-translational stabilisation and then acts as a master transcriptional regulator to induce the expression of many target genes. Analysis of tumours using immunohistochemistry and ELISA-based methods has shown that as many as 50% of human cancers show elevated expression of p53 compared to normal surrounding tissues. In contrast, analysis at the RNA level has typically shown little variation with the exception of a rare class of breast cancers that show very low levels of mRNA (Wei *et al*, 2006). This picture is now changing rapidly with the discovery of p53 isoforms whose mRNAs show more variable patterns of expression. Quantitative analysis of large numbers of human tumours using panels of antibodies has allowed a variety of expression phenotypes to be classified, including post-translational modifications and examination of the expression of p53-induced gene products (Nenutil *et al*, 2005). The relationship between these expression patterns and the genetic mutations in the p53 pathway is not straightforward. For example, mutation of the *p53* gene itself is not enough to confer stability and the WT protein can be stabilised by overexpression of the murine double minute (Mdm)2 or Mdm4 protein or by exposure to a DNA-damaging environment (Lane and Hall, 1997). Similarly, the relationship between p53 mutation and cancer prognosis has proved complex. In meta-analysis, *p53* mutation is generally associated with poor survival. However, the effect of p53 status on the response to therapy is variable. In some tumours, activation of wild type (WT) p53 seems important for the induction of the antitumour response. In other cases, the cell cycle arrest induced by p53 protects tumour cells against the therapeutic drug and tumours with mutant p53 show a better therapeutic response (Bertheau *et al*, 2002).

p53 is under precise control by the protein Mdm2 (Hdm2), which acts as an E3 ligase and targets p53 for ubiquitin-dependant degradation, acting as a critical negative regulator (Bond *et al*, 2005). Besides Mdm2 (Hdm2), the proteins Mdm4 (Hdm4, MdmX, HdmX) and Arf (p14^ARF^ in humans and p19^ARF^ in mouse) also play an important role in controlling p53 stability. Mdm4 is a structural homologue of Mdm2, while Arf is a tumour suppressor that interacts with Mdm2 and inhibits p53 degradation, thereby stabilising it. While these proteins have helped to reveal the different layers of regulation of p53 degradation, there are several questions concerning their interactions that remain unresolved. Since the loss or mutation of p53 function is associated with increased cancer susceptibility, reactivating WT p53 in those tumours where its function has been suppressed has been a target of several small molecule inhibitors currently being studied or being evaluated in the clinic (Figure 1). In a second approach, a search has been made for molecules that can activate the function of mutant p53 proteins. A third strategy gaining considerable attention advocates the use of an S or G2 phase-specific cytotoxic anticancer drug in combination with a p53-activating molecule for the treatment of tumours with mutant p53. In this approach, the molecule activating WT p53 acts as a protective agent, reducing damage to normal tissue by inducing cell cycle arrest, and thus increasing the therapeutic index of the S or G2 phase-inhibiting compound. Such a concept may, for example, protect against the bone marrow loss, hair loss and gastric problems that limit current cytotoxic therapy. In this review, we discuss some of the recent developments in the applications and therapeutic potential of targeting the p53 pathway in cancer therapy. In particular, we will concentrate on two classes of compounds that are either in clinical trial or close to trial that target the stability and activity of p53.

## 

### Mdm2 (Hdm2)/Mdm4 (Hdm4, MdmX, HdmX)

The ubiquitin ligase Mdm2 prevents the interaction of p53 with the basal transcription machinery and ubiquitinates it for proteasomal degradation. p53 in turn controls the expression of Mdm2, thus creating a negative feedback loop. In addition, Mdm4 is also a specific inhibitor of p53. These two are clinically important as their levels are amplified in at least 10–20% of human cancers. The critical importance of Mdm2 and Mdm4 as negative regulators of p53 has been established not only biochemically but also genetically. Deficiency in either protein results in embryonic lethality, but this lethality is totally dependant on p53. Thus, for example, *p53*−/−:*Mdm2*−/− mice are viable, while *p53*+/−:*Mdm2*−/− mice are not and the same is seen for Mdm4−/− mice.

This has naturally led to an intense exploration of WT p53 activation by downregulating Mdm2 function (Coutts and La Thangue, 2007; Toledo and Wahl, 2007; Vassilev, 2007). The three main approaches include repressing the expression of Mdm2, inhibiting the p53–Mdm2 interaction and blocking the ubiquitin ligase activity of Mdm2. Blocking Mdm2 expression with antisense oligonucleotides has been established successfully in cells in tissue culture and also in mouse models and warrants further investigation. More recently, small molecule inhibitors of transcription (the cyclin-dependent kinase (CDK) inhibitors) have been found to block Mdm2 expression and activate p53 as discussed later.

### Targeting the p53–Mdm2 interaction

Targeting the p53–Mdm2 interaction with small molecule inhibitors as a potential therapeutic strategy to activate WT p53 has been reviewed earlier (Vassilev, 2007). Proof of concept for this was established using synthetic peptides and protein aptamers, while nutlins were identified as the first selective Mdm2 inhibitors that displaced p53 from Mdm2 (Chene, 2003; Vassilev, 2007) The nutlins have since then been shown to inhibit tumour growth and cause tumour shrinkage at non-toxic doses in mouse models (Vassilev *et al*, 2004; Tovar *et al*, 2006). These were the first studies providing proof of concept of exploiting this approach of p53 activation as a potential cancer therapeutic. They have also shown the capacity of WT p53 activation to protect normal cells from S and G2 phase-specific cytotoxic drugs. Since then, several other studies have targeted p53–Mdm2 binding to identify novel compounds, including peptidomimetics, such as *β*-peptides, *β*-hairpin, chlorofusin, terphenyls, tryptophan-based peptides, stapled peptides and small molecules such as chalcones, aryl sulphonamides, 1,4-benzodiazepine-2,5-diones, isoindolinones, etc. (Dudkina and Lindsley, 2007).

Shaomeng Wang's lab used structure-based design to identify a particularly promising set of spiro-oxindoles and quinolinols as new potent and specific non-peptide, small molecule Mdm2 antagonists (Ding *et al*, 2006; Lu *et al*, 2006). Both these classes of compounds have shown promising results in cell lines, inducing cellular responses in a p53-dependent manner. Their *in vivo* activity and also their detailed molecular mechanism and biological activity are yet to be characterised. However, both these classes are promising leads for further refinement and development against WT p53 and Mdm2 as ‘druggable’ targets. Both these classes of compounds also further highlight the potential of structure-based drug design in identification of new compounds that target the p53 pathway.

Interestingly, 10 of the 13 amino acids of the binding cleft of Mdm2 that mediates its interactions with WT p53 are conserved in Mdm4 and yet nutlin does not potently inhibit the p53–Mdm4 interaction. In addition, the interaction between the transactivation domain of p53 and the N-terminal domain of Mdm2 is also known to modulate the interaction between an acidic domain of Mdm2 and the DNA-binding domain of p53; whether this interaction (a) extends to interactions with Mdm4 and (b) can be targeted for increasing the stability of WT p53, remains to be seen. A dynamic model integrating the specific and complementary roles of Mdm2 and Mdm4 has been proposed (Toledo and Wahl, 2007). These interactions are modulated by a range of post-translational modifications such as phosphorylation, acetylation, ubiquitination, sumoylation, neddylation and glycosylation and provide an avenue for further development of inhibitors based on mechanistic details of how these modifications stabilise/destabilise the p53–Mdm2/Mdm4 system.

Yet another compound that inhibits the WT p53–Mdm2 interaction identified through a chemical library screen is called RITA (reactivation of p53 and induction of tumour cell apoptosis) (Issaeva *et al*, 2004). RITA has been suggested as another non-genotoxic method by which p53 transcription and also p53-dependent apoptosis may be activated. Unlike the previously characterised Mdm2 antagonists, RITA has been proposed to inhibit the p53–Mdm2 interaction by binding to WT p53. p53-dependent antitumour activity was also demonstrated by systemic administration of RITA in severe combined immunodeficiency mice with HCT116 p53+/+ and p53−/− xenografts, further suggesting its potential for development as a therapeutic strategy to activate WT p53. However, more detailed studies of the mechanism of action of RITA are warranted since conflicting NMR structural studies later reported that RITA does not inhibit the p53–Mdm2 interaction (Krajewski *et al*, 2005).

High-throughput screening of E3 ligase inhibitors led to the identification of the HL198 compounds that inhibit Mdm2 autoubiquitination and thereby activate WT p53 transcriptional activity and also p53-dependent apoptosis (Yang *et al*, 2005). However, even though these compounds provide proof of principle that this could be a potential strategy to target the p53 pathway, further studies need to be performed to identify more selective and potent analogues of the HL198 compounds before they can be taken forward as a treatment strategy.

Deubiquitinating enzymes (DUBs) have been established as yet another critical modulator of p53 stability, the most notable example being HAUSP (herpesvirus-associated ubiquitin-specific protease), which has been shown to deubiquitinate p53, Mdm2 and Mdm4 in a concentration-dependent manner (Li *et al*, 2002; Meulmeester *et al*, 2005). More recently, USP2a (ubiquitin-specific protease 2a) has been identified as a novel DUB that selectively targets Mdm2, unlike HAUSP, and thereby offers another potential approach of therapeutic intervention to reactivate WT p53 (Stevenson *et al*, 2007). Future studies will further address the importance of these DUBs as clinical targets and also the selectivity of their use based on tumour subtypes. Further study of the degradation of p53 has identified additional E3 ubiquitin ligases and E2 proteins that can also modulate p53 levels. These additional proteins are listed in Table 1. While the genetic definition of the importance of these additional proteins lacks the precision achieved in the Mdm2/Mdm4 and Arf systems, each could represent a valid target for drug discovery. It will, however, be critically important to further validate these targets using genetic and aptamer strategies in whole animals, and this could also rest upon their selective overexpression in particular tumour types.

In addition, other identified cellular factors that modulate the p53–Mdm2 interactions are gankyrin, L11, p14arf, p300, YYI and more recently, the ribosomal protein S7, which stabilises WT p53 by interacting with the p53–Mdm2 complex and preventing the ubiquitination of p53 (Chen *et al*, 2007). Understanding and exploiting the mechanisms of action of these natural inducers of WT p53 stability offer promising avenues for therapy.

A related attempt that seems hopeful is to reactivate mutant p53. A majority of the mutations in *p53* are located in the DNA-binding domain, with complex functional consequences. These mutations can affect the thermodynamic stability, folding rates of p53 and the interactions of p53 with DNA as well as with other partner proteins (Joerger and Fersht, 2007). Discovery of second site mutations that restore the activity of some of these mutants has provided clues for the restoration of activity by the use of small molecules. The idea is based on the notion that the small molecule will bind preferentially to the ‘properly’ folded state of p53. This has led to the identification of a peptide and some small molecules, including CP-31398, ellipticine, WR1065, MIRA-1 (mutant p53-dependent induction of rapid apoptosis) and their derivatives that were found to rescue WT activity of several misfolded mutants. None of these molecules, however, yet show a complete link between mechanism and *in vivo* activity. For the peptide classes, the physical and biological evidence is clear but the *in vivo* activity is not. For MIRA-1, xenograft activity is established, but the biochemical target and physical specificity of this tiny molecule is unresolved.

### CDK inhibitors

Several CDK inhibitors are in various stages of research and development as anticancer agents. R-roscovitine and olomoucin, which were developed as CDK inhibitors, have been shown to activate p53 by inhibiting expression of Mdm2 and thereby blocking p53 degradation by Mdm2 (Lu *et al*, 2001). However, neither the p53–Mdm2 binding nor the nucleocytoplasmic shuttling of p53 or Mdm2 is directly affected by these inhibitors. Flavopiridol, another CDK inhibitor, has also been shown to activate WT p53 by initially inhibiting Mdm2 (Demidenko and Blagosklonny, 2004). These studies show the potential of CDK inhibitors to affect p53 activation and degradation by downregulating Mdm2 (Lu *et al*, 2001). Another proposed model suggests the nucleolus as a stress sensor and suggests that disruption of the nucleolus by the CDK inhibitors releases key ribosomal protein inhibitors of Mdm2 and prevents WT p53 degradation (Rubbi and Milner, 2003). While this has been proposed as a unifying model to explain activation and stabilisation of WT p53 by various agents, further detailed *in vivo* studies in animal models and ultimately the response seen in patients will have to be further evaluated for CDK inhibitors to be used as standard anticancer agents. While in cancer cell lines the apoptotic response to CDK inhibitors is only partially p53 dependant, studies using the zebrafish show a remarkable p53 dependence of the apoptotic response in the whole organism system. Also, the role of the CDK inhibitors as p53-inducing cytoprotective agents has not yet been investigated.

### Drug synergy and biomarkers of response

A common theme emerging in cancer treatment is that combination therapy may be the ideal way to combat the problems of drug-related toxicity and resistance. While most attention has been given to using drug combinations to activate distinct pathways, it is also possible to imagine developing combinations of molecules that focus on the activation of a single specific pathway. One might see synergy by combining different p53 activators, for example, recent studies in our lab (Cheok *et al*, in press) show striking synergistic non-genotoxic activation of WT p53 upon combination of nutlin-3, R-roscovitine and DRB (5,6-dichloro-1-*β*-D-ribofuranosylbenzimidazole) at much lower concentrations than that required to activate WT p53 by individual drugs alone. Among the various WT p53 activators, nutlins have probably been the most studied in human cancers. While most of the efforts have focused on using nutlin-3 as a single agent for applications in oncology, some recent studies have also combined nutlin-3 with chemotherapeutic agents and seen potentiation of activity of these chemotherapeutics *in vitro*, in acute myelogenous leukaemia (Kojima *et al*, 2005), multiple myeloma (Stuhmer *et al*, 2005) and chronic lymphocytic leukaemia (Coll-Mulet *et al*, 2006). These initial studies definitely look promising and warrant more detailed studies in animal models. Their success would ultimately be evaluated by the response rates seen in patients in the clinic, either as single agents in certain cases or in combination with standard chemotherapy in others. Several tumour types have overexpression or aberrant expression of Mdm2, thereby lowering p53 activity. Hence, Mdm2 inhibitors would be most effective in treatment of such tumours to activate p53. This also suggests that besides the p53 status in tumours (since most of these antagonists are dependent on the WT p53 status of tumours), the Mdm2 status in tumours could be another useful biomarker of response in evaluating potential efficacy of treatment. The choice of tumour types and relevant patient population selection would be the key in the evaluation of the success of these compounds activating WT p53 by controlling its degradation and stability. A key issue to be resolved is the cellular basis of the variant response to WT p53 activation. Thus, in some cells WT p53 activation induces senescence while in others it induces apoptosis and in yet others reversible cell cycle arrest. To be effective as a therapeutic strategy, p53 activators will need to induce cell cycle arrest in normal tissues but apoptosis or senescence in tumour cells. The therapeutic activity of the nutlins in mouse models suggests that activating WT p53 by inhibiting Mdm2 may produce such an outcome. Recent elegant studies by Lowe's group (Xue *et al*, 2007) and Jacks' group (Ventura *et al*, 2007) have shown tumour regression in various tumour types in mice by restoring WT p53 function alone. In the case of the Jacks' model, this was due to tumour-specific apoptosis while in the Lowe's model the induction of senescence was critical. However, neither of these models addressed the issue of therapeutic index since the WT p53 function was restored only to tumour cells and not to normal cells (Ventura *et al*, 2007; Xue *et al*, 2007). In a very revealing study, Bernards and co-workers used siRNA approaches to search for genes whose inactivation would confer resistance to nutlin (Brummelkamp *et al*, 2006). Satisfyingly, p53 was identified, but provocatively they also showed that inactivation of the p53BP1 (p53 binding protein 1) DNA damage response gene was also effective. Thus, an attractive model is that the constitutive stress environment in tumour cells as opposed to normal cells influences the p53 response towards senescence or apoptosis as opposed to growth arrest. Such a dual-signal molecule can explain the tumour selectivity of the cytotoxicity of WT p53-activating molecules and the therapeutic index of the nutlins. This model also would further support the use of WT p53-activating molecules as cytoprotectives.

## CONCLUDING REMARKS AND PERSPECTIVES/ FUTURE PROSPECTS

Targeting p53 to combat cancer is definitely a very attractive strategy with significant advances made in the recent years. While several lead compounds have been identified that activate WT p53 and affect its degradation and stability, it must be noted that all these compounds affect both normal and tumour cells. They are still promising in light of knowledge that tumour cells are more sensitive to apoptosis than normal cells, but selectivity of compounds is perhaps the critical aspect that should be addressed in further development of therapeutic strategies. Combination therapy is emerging as a key factor, and development of non-genotoxic combinations seems very promising for tackling the problems of toxicity and resistance. The next few years hold the possibility of fulfilling the translation of the discoveries in the basic biology of the p53 system into patients' benefit.

## Figures and Tables

**Figure 1:**
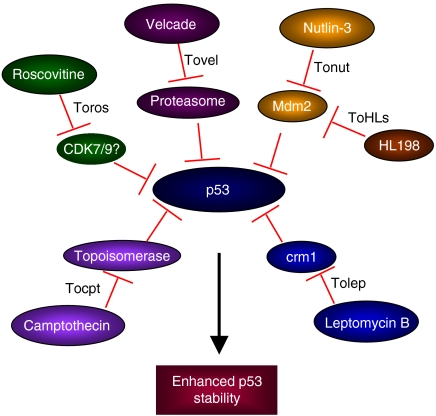
Small molecules that modulate p53 degradation and stability.

**Table 1:** Enzymes that modulate p53 stability

**E3 ligases**	**E2 ligases**	**DUBs**	**Others**
Mdm2	Ubc9	HAUSP	YY1
Cop1	Ubc13	(USP7)	LZAP
PIRH2	UbcH5B/C	USP2a	Prolyl isomerase Pin1
ARF-BP1			Ribosomal proteins
WWP1			L11, L23
E6/E6-AP			
TOPORS			
CUL4			
p53RFP			
STUB1 (CHIP)			

ARF-BP1=alternative reading frame-binding protein 1; CHIP=carboxy terminus of Hsp70-interacting protein; COP1=constitutively photomorphogenic 1; CUL4=cullin 4; HAUSP=herpesvirus-associated ubiquitin-specific protease; LZAP=leucine-zipper-containing ARF-binding protein; Mdm2=mouse double minute 2; PIRH2=p53-induced RING-H2 protein; p53RFP=p53-inducible RING-finger protein; STUB1=STIP1 homology and U-box containing protein 1; TOPORS=topoisomerase I-binding RS protein; Ubc=ubiquitin-conjugating enzyme; USP=ubiquitin-specific protease; YY1=Yin Yang 1.
